# The contribution of serum hepatitis B virus load in the carcinogenesis and prognosis of hepatocellular carcinoma: evidence from two meta-analyses

**DOI:** 10.18632/oncotarget.10335

**Published:** 2016-06-30

**Authors:** Xueqin Chen, Fan Wu, Yanmei Liu, Jiao Lou, Beibei Zhu, Li Zou, Wei Chen, Jing Gong, Ying Wang, Rong Zhong

**Affiliations:** ^1^ Department of Epidemiology and Biostatistics, School of Public Health, Tongji Medical College, Huazhong University of Science and Technology, Wuhan, Hubei, China; ^2^ Department of Medical Quality Management, Jiangxi Cancer Hospital, Nanchang, Jiangxi, China; ^3^ Abdominal Surgery Department, Cancer Hospital, Peking Union Medical College, Chinese Academy of Medical Sciences, Beijing, China; ^4^ Department of Infectious Diseases, Tongji Hospital, Tongji Medical College, Huazhong University of Science and Technology, Wuhan, Hubei, China; ^5^ Department of Virology, Wuhan Centers for Disease Prevention and Control, Wuhan, Hubei, China

**Keywords:** HBV DNA level, hepatocellular carcinoma, hepatocellular carcinoma recurrence, meta-analysis

## Abstract

**Background and Aim:**

The meta-analysis aimed to quantify and summarize the contribution of serum hepatitis B virus (HBV) DNA load in the carcinogenesis and prognosis of hepatocellular carcinoma (HCC).

**Results:**

Nine independent studies with a total of 1162 cases and 9365 participants on risk of HCC and seventeen studies with 1342 cases and 2891 participants on recurrence of HCC were finally included. The non-liner dose-response association between HBV DNA level and HCC risk was observed, with *P* value equal to 0.02 for linear test. Compared with 2 log_10_copies/ml HBV DNA level carriers, the summary relative risk of HCC were 1.65(95% CI: 0.94–2.92) for 4.5 log_10_copies/ml, 2.20(95% CI: 1.00–4.85) for 5.5 log_10_copies/ml, 3.06(95% CI: 1.11–8.44) for 6.5 log_10_copies/ml. Moreover, individuals with high viral load (HBV DNA levels > 10^5^copies/ml) presented significant association with increased risk of HCC recurrence, with the pooled RR of 1.69 (95% CI: 1.49–1.92).

**MATERIALS AND METHODS:**

Pertinent studies were identified by searching PubMed, Embase and ISI Web of science databases up to January 2016 and by reviewing the references of retrieved articles. The dose-response meta-analysis was precisely performed to calculate the summary relative risks (RRs) by quantizing the association between HBV load and risk of HCC. Besides, the contribution of HBV load on recurrence of HCC was further clarified by general meta-analysis.

**Conclusions:**

These findings indicated a non-linear dose-response relationship between serum HBV DNA level and risk of HCC, and confirmed the significant contribution of serum HBV DNA level in the prognosis of HCC.

## INTRODUCTION

Hepatocellular carcinoma (HCC), a major health challenge worldwide, is the sixth most common cancer [1], and the third leading cause of cancer mortality [2]. The biological mechanisms of pathogenesis and prognosis for HCC were strongly influenced by diverse etiologies involving in both host and viral factors. Among the major risk factors for hepatocarcinogenesis, hepatitis B virus (HBV) infection is of particular importance and has been designated as the major etiology of HCC. Throughout the world, approximately 2 billion people suffer from infection of HBV, which would cause a broad spectrum of liver diseases ranging from asymptomatic carrier, chronic hepatitis and liver cirrhosis to HCC [3]. Etiologically, more than 50% of HCC cases worldwide are attributable to persistent infection of HBV, and reach to 75%–85% of HCC cases in developing countries [4].

HBV DNA load, a sign of active virus replication, which are used to assess the efficacy of the antiviral therapy, has been gradually estimated as an important predisposing factor of HCC [5–7]. More importantly, greater focus has been placed recently on clarifying the role of antiviral therapy in the carcinogenesis of HCC [8, 9]. However, before figure out the relationship between antiviral therapy and risk of HCC, the detailed effect of HBV DNA load on risk of HCC should be firstly established. Meanwhile, growing evidence from prospective epidemiologic studies is accumulating rapidly to support a very strong relationship between HBV viral load and risk of HCC. However, the controversial results were yielded by recent previous studies for detailed shape of association between HBV DNA load and risk of HCC. Some previous studies demonstrated that risk of HCC only started to increase significantly at the HBV DNA level of 10^4^ copies/ml [6, 10, 11], in contrast to the findings of Sun *et al.* [12]. Additionally, some previous studies observed a linear dose-response relationship between HBV DNA load and risk of HCC, but a nonlinear association was also found by other studies which addressed the hazard ratio increased with HBV DNA level in the range of 10^4^ to 10^6^ copies/ml but abruptly decreased with level > 10^7^ copies/ml [13, 14]. Therefore, it has been strongly proposed that the detailed shape of quantitative relationship between HBV DNA level and HCC risk should be systematically evaluated by dose-response meta-analysis to determine the non-linear or linear dose-response association. Moreover, the clarification of the detailed relationship will provide valuable clues to elucidate the important roles of the HBV DNA level in hepatocarcinogenesis.

In addition, the prominent characteristic for HCC with dismal prognosis is extremely pernicious although the treatment options have been stepped forward. HCC recurrence is a main obstacle of HCC prognosis, remaining a paramount cause of death in HCC patients [15]. Despite surgical or local regional therapies, the recurrence rates can be as high as 50% at 2 years [16, 17]. Consequently, it is of great importance to identify and improve modifiable risk factors to prevent from HCC by considering the malignancy and high recurrence rates of HCC. Theoretically, HCC recurrence may partly be attributable to metachronous multicentric carcinogenesis and *de novo* HCC [18], which could be enhanced by HBV load. Some of previous studies [19, 20] showed that high viral load (HBV DNA levels > 10^5^copies/ml) promotes the recurrence of HCC, but this significant associations were not estimated by Chung *et al.* [21] and Mathews *et al.* [22]. Considering the controversial results which have been yielded by previous studies, and the limited power which was estimated by the studies with relatively small sample size, the obscure relationship between HBV load and recurrence of HCC was urgent to be clarified by a meta-analysis with enough power via combining the basis of published data.

Since HBV DNA load can be alleviated by the treatment of anti-viral agents, it seems important to clarify the exact effect of HBV DNA level on HCC risk and recurrence. The settlement of this problem might somewhat influence the decision of clinically anti-viral treatment for patients with high HBV DNA level. Here we performed a dose-response meta-analysis to discern the potential quantitative association between HBV load and HCC risk. Meanwhile another meta-analysis was also conducted to clarify the controversial relationship between HBV load and HCC recurrence.

## RESULTS

### Literature searches

**Figure 1:**
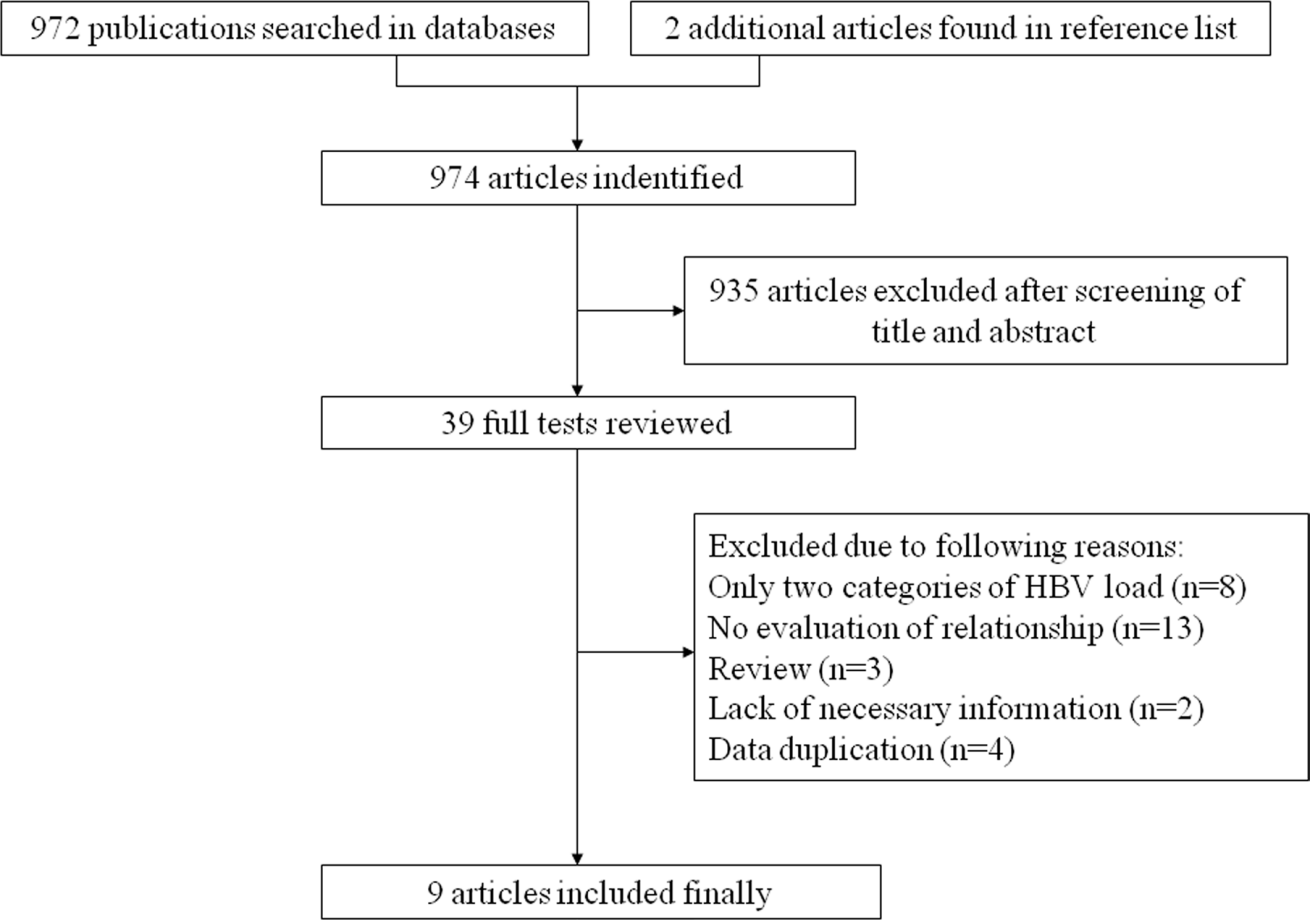
The flow chart of literature search on HBV DNA level and HCC risk

**Figure 2:**
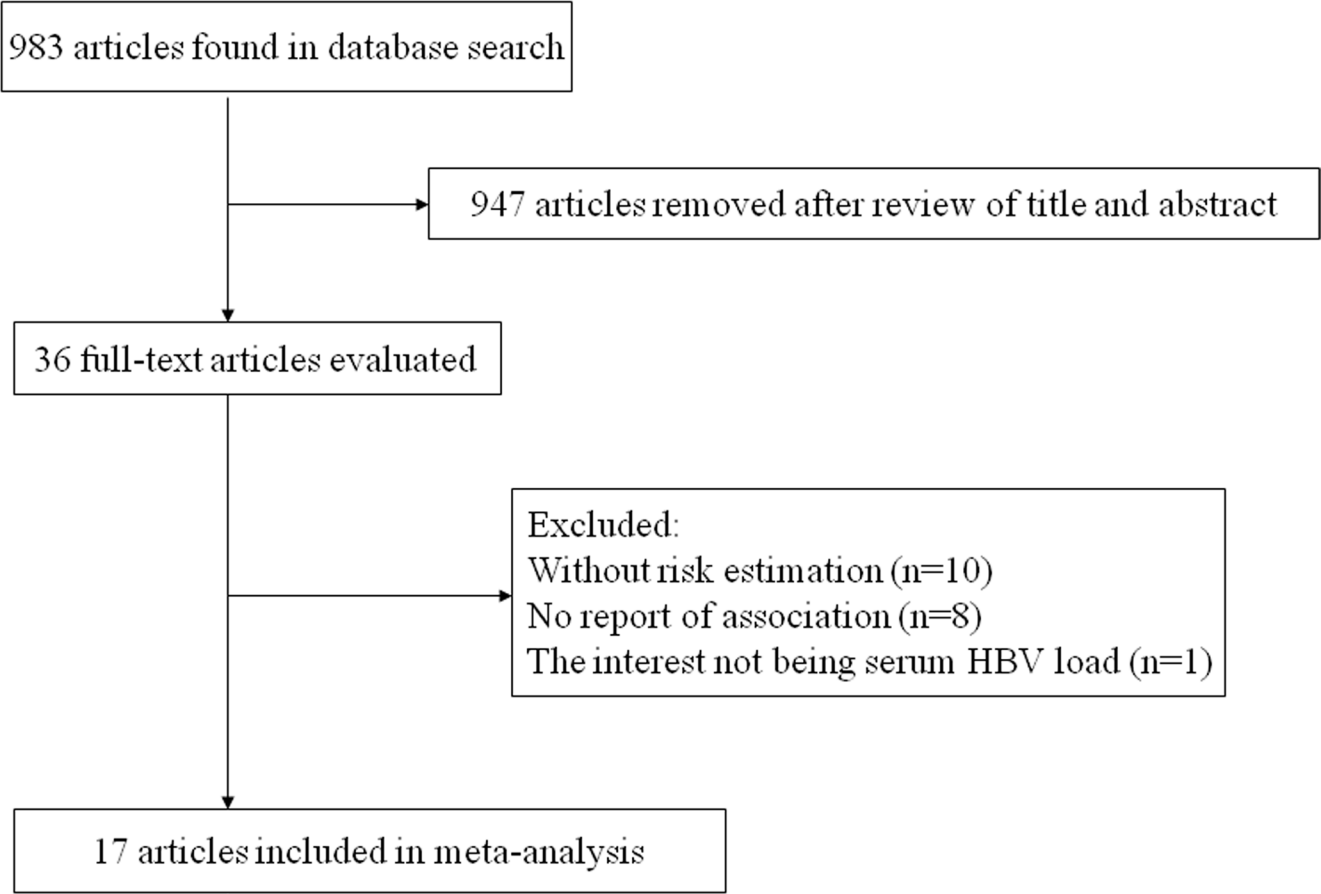
The selection procedure of studies on the HCC recurrence

The flow chart of literature search on HBV DNA level and HCC risk was shown in Figure 1. A total of 972 publications were totally identified and 935 were excluded after review of title and abstract. After examining the remaining 37 full texts, 30 articles were further excluded owing to the following reasons: only two categories of HBV DNA level (*n* = 8), no evaluation of the relationship between HBV DNA level and HCC risk (*n* = 13), review articles (*n* = 3), lack of necessary information (*n* = 2), and data duplication (*n* = 4). Besides, two additional articles [14, 23] were added by looking through the references of the retrieved articles. Therefore, nine studies [5, 6, 10, 11, 14, 23–25] were included in the final analysis. Similarly, Figure 2 showed the selection procedure of studies on the HBV load and HCC recurrence. The primary searches yielded 983 items, of which 947 articles were removed after scanning the title and abstract. After review of 36 full texts, 10 studies without risk estimation and 8 studies with no report of association between HBV load and HCC recurrence were further excluded. We also removed one report since its interest was HBV DNA titer in surrounding liver. Finally, a total of 17 articles [1, 19–22, 26–37] were pooled into analysis.

### Study characteristics

The detailed information extracted from included studies was shown in Table 1 and Table 2. The dose-response meta-analysis contained 5 case-control studies with 657 cases and 1595 objects, and 4 cohort studies with 505 HCC patients and 6765 participants totally. All of the studies were conducted in Asia. Furthermore, the meta-analysis on HCC recurrence included 1342 cases and 2891 participants. Among the 17 articles, 9 studies were conducted in HCC patients with treatment of tumor resection, and 6 studies in patients with local ablation.

**Table 1:** Characteristics of studies on HBV DNA level and HCC risk

Study	Publication year	Study location	Study design	No of cases	Sample size/person years	HBV DNA level (log_10_copies/ml)	Adjusted RR	95% CI	Adjustment variables
Yu	2005	Taiwan	Case-control	154	470	UD	1.00		Age, date of blood collection, ethnicity, history of cigarette smoking and alcohol drinking
						3.62–4.22	1.07	0.44–2.60	
						4.23–4.90	2.54	1.16–5.59	
						4.91–5.90	2.44	1.12–5.28	
						5.91–10.81	7.26	3.54–14.89	
Liu	2008	China	Case-control	170	446	UD	1.00		Age, cigarette smoking, alcohol consumption, and family history of chronic liver diseases
						2.69–3.99	0.47	0.17–1.256	
						4.00–4.99	2.83	1.24–6.49	
						5.00–5.99	48.40	14.39–162.79	
						6.00–6.99	42.25	14.78–120.75	
						≥ 7.00	14.82	6.99–31.41	
Xu	2009	China	Case-control	155	310	< 3.00	1.00		None
						3.00–4.00	1.34	0.55–3.30	
						4.00–5.00	2.39	1.13–5.05	
						5.00–6.00	2.61	1.18–5.77	
						6.00–7.00	2.05	0.97–4.33	
						≥ 7.00	1.28	0.46–3.54	
Asim	2010	India	Case-control	88	188	≤ 4.50	1.00		Age, sex, HBeAg status, genotype, mutations in the X region, mutations in the BCP region, mutations in the precore region, mutations in the core region
						4.50–6.50	2.27	1.13–4.58	
						> 6.50	2.62	1.06–6.45	
Zhou	2012	China	Case-control	90	180	< 3.00	1.00		None
						3.00–4.00	1.38	0.54–3.50	
						4.00–5.00	3.67	1.36–9.89	
						5.00–6.00	5.30	1.85–15.28	
						≥ 6.00	3.03	1.14–8.04	
Chen	2006	Taiwan	Cohort	164	41779	< 2.48	1.00		Sex, age, cigarette and alcohol use, HBeAg, ALT level, and cirrhosis at entry
						2.48–4.00	1.10	0.25–2.30	
						4.00–5.00	2.30	1.10–4.90	
						5.00–6.00	6.60	3.30–13.10	
						> 6.00	6.10	2.90–12.70	
Wong	2010	Hongkong	Cohort	45	424	< 4.50	1.00		None
						4.50–6.50	1.97	0.80–4.83	
						> 6.50	2.88	1.23–6.77	
Wong	2010	Hongkong	Cohort	105	1005	< 4.50	1.00		Age, sex, albumin, bilirubin, ALT, radiologic cirrhosis, antiviral therapy
						4.50–6.50	1.83	1.03–3.25	
						> 6.50	3.40	1.97–5.87	
Tseng	2012	Taiwan	Cohort	191	39427	< 3.00	1.00		None
						3.00–4.00	0.90	0.40–1.90	
						4.00–5.00	2.00	1.00–3.90	
						5.00–6.00	4.10	2.10–8.00	
						> 6.00	5.10	2.80–9.20	

Abbreviations: HBV, hepatitis B virus; HBeAg, hepatitis B e antigen; HCC, hepatocellular carcinoma; RR, relative risk; CI, confidence interval; UD, undetectable; BCP, basal core promoter; ALT, alanine aminotranferase.

**Table 2:** Characteristics of studies on HBV load and recurrence of HCC

Author	Publication year	Study location	Treatment	No of cases	No of participants	Adjusted RR	95% CI	Adjustment for covariates
Jang	2007	Korea	TACE	32	62	3.77	1.70–8.38	HBeAg status, Child-Pugh classification, No. of tumors, lamivudine use
Kim	2008	Korea	Resection	75	157	1.61	1.01–2.55	Tumor size, tumor number, vascular invasion, and grades
Chuma	2009	Japan	Resection or RFA	38	64	2.67	1.31–5.47	Gender, age, HBeAg status, ALT, Platelet count, PT, Albumin, Bilirubin, Liver fibrosis, tumor differentiation, AFP, PIVKA-II, tumor size tumor number initial treatment
Wu	2009	Taiwan	Resection	39	92	2.55	1.04–6.24	Ishak activity, multinodularity, ICG-15
Qu	2010	China	Resection	183	317	2.11	1.48–3.00	Alpha-fetoprotein, microvascular, invasion, postoperative IFN-α treatment
Liang	2010	China	Resection	38	64	1.80	0.80–4.11	None
An	2010	Korea	Resection	95	188	1.80	0.85–3.80	ALT, Child-Pugh class B, HBeAg seropositivity, vascular invasion, cirrhosis, tumor size
Goto	2011	Japan	RFA	42	69	2.67	1.16–6.14	Albumin, Platelet count, prothrombin activity, Child-Pugh Class, number of nodules
Chung	2011	Korea	Local ablation	63	140	1.02	0.61–1.71	None
Li	2011	China	OLT	43	148	2.45	1.10–5.45	Age, gender, number of nodules, the largest nodule size, exceeding Milan criteria, vascular invasion, tumor differentiation, preoperative serum AFP level, HBV recurrence
Xia	2012	China	RFA	67	152	1.73	0.88–3.40	None
Mathews	2012	Korea	Resection	99	247	1.01	0.50–2.02	Age, gender, serum AFP, serum ALT, advanced MELD score, tumor size, microvascular invasion, advanced CLIP stage, E-S grade
Su	2013	Taiwan	Resection	208	333	1.43	1.05–1.95	GGT, macroscopic venous invasion, microscopic venous invasion
Kim	2014	Korea	RFA	70	300	1.55	0.79–3.03	None
Sohn	2014	Korea	RFA	89	170	1.57	1.02–2.41	Multinodularity, BCLC stage
Sohn	2014	Korea	Resection	61	188	2.77	1.62–4.73	Age, sex, Platelet, ALT, AFP, DCP, HBeAg,
Hung	2016	Hong Kong	Resection	100	200	1.67	1.09–2.57	AFP, tumor size, antiviral treatment, tumor differentiation, lymphovascular permeation, microsatellite lesions

Abbreviations: HBV, hepatitis B virus; HCC, hepatocellular carcinoma; RR, relative risk; CI, confidence interval; TACE, transarterial chemolipiodoization, HBeAg, hepatitis B e antigen; AFP, alpha-fetoprotein; RFA, radiofrequency ablation; ALT, alanine aminotranferase; PT, prothrombin time; PIVKA-II, protein induced by vitamin K absence or antagonist II; ICG-15, indocyanine green 15-min retention rate; OLT, orthotopic liver transplantation; MELD, Model for End-Stage Liver Disease; BCP, basal core promoter; CLIP, Cancer of the Liver Italian Program; E-S grade, Edmondson-Steiner grade; GGT, γ-glutamyl transpeptidase; BCLC, Barcelona Clinic Liver Cancer; DCP, des-γ-carboxy prothrombin.

### Dose-response meta-analysis of association between HBV DNA level and HCC risk

**Figure 3:**
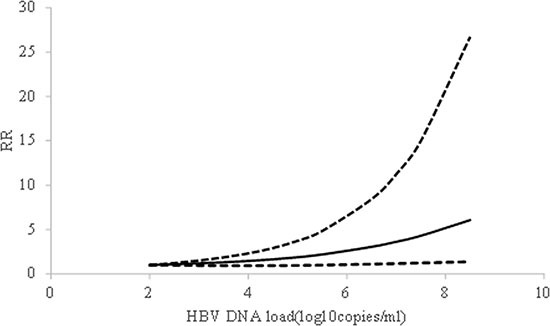
The shape of association between HBV DNA level and HCC risk, with restricted cubic splines in random-effects dose-response model The solid line and the short dash line represent the estimated relative risks and corresponding 95% CIs, respectively.

We found evidence of non-linear association between HBV DNA level and risk of HCC (*P* = 0.02 for non-linearity). Compared with 2 log_10_copies/ml HBV DNA level carriers, the summary relative risk of HCC were 1.65 (95% CI: 0.94–2.92) for 4.5 log_10_copies/ml, 2.20 (95% CI: 1.00–4.85) for 5.5 log_10_copies/ml, 3.06 (95% CI: 1.11–8.44) for 6.5 log_10_ copies/ml. Random-effects model was applied, since moderate heterogeneity between studies was observed (*I*^2^ = 56%) (Figure 3). In the sensitivity analysis, the pooled RRs did not alter essentially, indicating that the summary estimate were reliable and stable. Additionally, no significant publication bias was detected by Egger's test (*P* = 0.76).

### Overall meta-analysis of relationship between HBV load and recurrence of HCC

**Figure 4:**
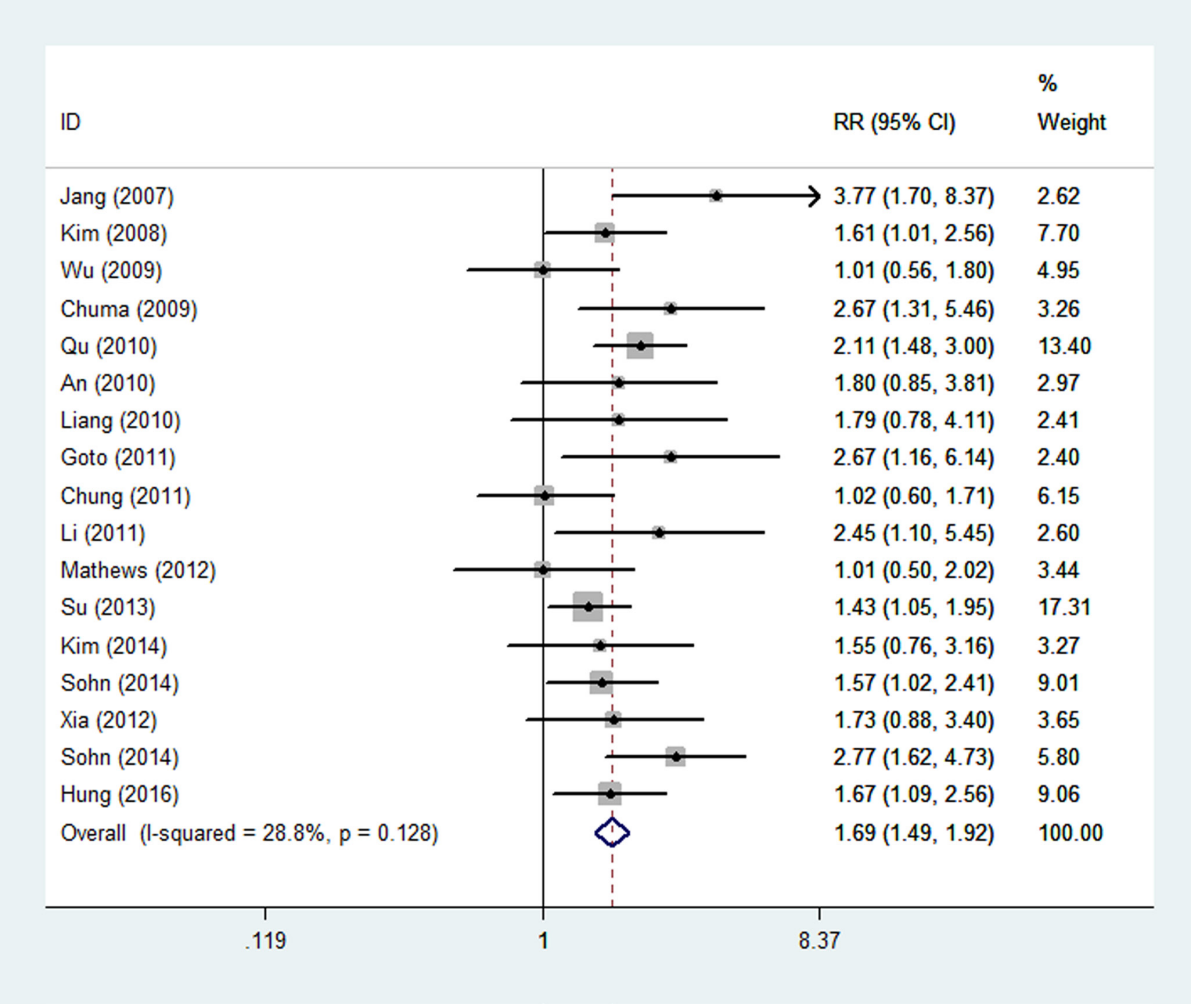
The forest figure of overall meta-analysis on HBV load and recurrence of HCC

The overall meta-analysis showed that there was a significant association between HBV DNA load and risk of HCC recurrence, with pooled RR of 1.69 (95% CI: 1.49–1.92) (Figure 4), using fixed-effects model with mild heterogeneity observed (*I*^2^ = 28.8%). Sensitivity analysis indicated that the summary estimate was not influenced by any single study. No evidence of statistically significant publication bias was identified by Egger's test (*P* = 0.33).

### Stratified analysis of relationship between HBV load and HCC recurrence

Stratified analyses were performed to explore the source of heterogeneity and examine the stability of the primary results (Table 3). When we conducted stratified analysis according to median duration of follow-up, study style and primary treatment method, the contribution of HBV load to HCC recurrence for all subgroups was similar to that for the overall analysis. The heterogeneity between studies was not greatly reduced in the subgroups.

**Table 3:** Pooled RRs with 95% CIs for the association between HBV DNA load and HCC recurrence by stratified analysis

Stratified factors	No of reports	RR (95% CI)	*P* for heterogeneity	*I*^2^(%)	*P* for test
Median duration of follow-up					
≥ 36 months	3	1.62 (1.17–2.27)	0.29	19.4	0.004
< 36 months	14	1.81 (1.43–2.30)	0.02	50.7	0.000
Study style					
Prospective	8	1.73 (1.13–2.65)	0.01	65.2	0.011
Retrospective	9	1.83 (1.55–2.15)	0.46	0.0	0.000
Primary treatment					
Resection	9	1.71 (1.29–2.27)	0.02	56.1	0.000
RFA	5	1.73 (1.23–2.44)	0.12	43.3	0.002

## DISCUSSION

The current meta-analysis on risk of HCC indicated that there was a non-linear dose-response association between HBV DNA level and HCC risk. The RRs of HCC increased with HBV DNA lever, and leveled up at heavy HBV DNA load. Our study, to the best of knowledge, firstly explores the precise effect of HBV DNA level on HCC risk based on a non-linear dose-response relationship. Another overall meta-analysis confirmed the significant contribution of serum HBV DNA level in the recurrence of HCC, which would provide robust evidence for this controversial problem.

To date, the majority of studies have showed the risk of HCC increasing gradually with the augment of HBV load, albeit the positive cut off point were slightly different. However, Xu [14] and colleagues designed a matched case-control study and found that the RRs of HCC turned non-significant at high HBV DNA level. One potential explanation for this null association might be related to limited sample size in higher category of HBV DNA. In our study, the HCC risk started to increased significantly at HBV DNA level of 5 log_10_copies/ml, which was similar to the level suggested by current clinical practice guidelines for making management decisions in the care of chronic hepatitis B patients. Moreover, our study also showed that the patients after primary therapy with high HBV load are more likely to suffer the recurrence of HCC. The similar results were also demonstrated in stratified analyses. When we carried out the stratified analysis based on the median duration of follow-up, heterogeneity reduced obviously with *I*^2^ down to 19.4% in longer duration of follow-up studies. For HCC patients, most recurrence happened within two years, but there were still several smaller clusters of recurrences after two years [26]. Therefore, studies with shorter follow-up duration were unable to discover these patients who tended to late recurrence, leading to the different effect size derived from two subgroups. This may potentially explanted that some studies [30, 35] failed to disclose the positive relationship between HBV load and HCC recurrence. When stratified by study style, heterogeneity disappeared in the retrospective studies. Similar results were observed in both prospective and retrospective subgroups. The difference in effect size between the two groups could be probably caused by its own particular characteristics of each study design. Furthermore, stratified analysis was conducted by primary treatment. Heterogeneity remained unchanged or mildly increased after stratified by treatment. The results of subgroups were similar with overall analysis. Taken together antiviral therapy after tumor resection and RFA, when HBV DNA load is heavy, will produce substantially beneficial effect on the long-term prognosis of HBV related HCC patients.

The carcinogenic mechanisms of HBV are as follows. First, HBV DNA can integrate into hepatic cell randomly, which may induce chromosomal deletions and other genetic instability, contributing to HCC [38]. Moreover, cellular genes integrated with HBV DNA encode products with alterative structures, disrupting the crucial functions such as regulating cell signaling and controlling cell proliferation [39]. Second, HBx, a product encoded by HBV X gene, is involved to stimulate certain oncogenes and activate several signal pathways related to cell proliferation and cell apoptosis, leading to hepatocarcinogenesis [40]. Third, dynamic HBV replication may trigger chronic hepatopathy with gangrenous inflammation. As a result, a series of reaction from the inflammatory process can induce genomic changes and accelerate the development of HCC [41]. Although the precise mechanism of HCC recurrence related to HBV is not clearly elucidated, some previous studies have demonstrated that a high HBV viral load could cause malignant transformations in the remnant liver, which was similar with the mechanism of HCC, thus facilitating intrahepatic metastases or multicentric carcinogenesis, and then lead to recurrent tumor [42, 43].

The current study has its own strengths. First, we examined the shape of dose-response relationship between HBV DNA level and risk of HCC. Linear and non-linear trend were both evaluated to disclose the authentic association. Second, the significant relationship between HBV load and HCC recurrence was verified by meta-analysis, which could provide high statistical power by integrating independent studies with relative large sample size. Third, we evaluated the effect of HBV load not only on risk of HCC, but also on HCC recurrence, emphasizing the importance of antiviral treatment for chronic HBV infection patients to undergo a better outcome.

In spite of these advantages mentioned above, several limitations should also be acknowledged. First, the confounding factors which exited in every independent study cannot be settled in the meta-analysis, although most studies adjusted the main reported confounders or controlled them in the selection of participants. Second, we failed to evaluate the effect of high HBV load on HCC recurrence in subgroups of patients after transtarterial chemolipiodolization and orthotopic liver transplantation owing to the limited data. Third, even though we attempted to excavate the source of between-study heterogeneity existed in the dose-response meta-analysis, the results seemed to be regretful.

To our knowledge, the HBV infection is a global public health problem, particularly in epidemic regions such as East and Southeast Asia and sub-Saharan Africa [3, 44]. More importantly, the incidence of HBV related HCC has been increasing. The effect of high HBV load on both HCC risk and recurrence estimated in our meta-analyses would encourage the use of anti-viral therapy for both chronic hepatitis B and HCC patients to reduce HBV DNA level, aiming to decrease the incidence rate of HCC and improve the prognosis of HCC patients. Although the sound evidence of potential benefit of this strategy would come from the randomized controlled tests of antiviral agents, our study may provide robust etiological basis. In summary, this study not only identified the non-linear dose-response association between HBV DNA level and risk of HCC, but also revealed the contribution of high HBV load on HCC recurrence. Further studies with large sample size and well design are warranted to confirm our results.

## MATERIALS AND METHODS

### Search strategy and literature selection

We performed a literature search of PubMed, Embase and ISI Web of science databases from their inceptions to January 2016 using the key words “HBV DNA level or HBV load” and “hepatocellular carcinoma” for the dose-response meta-analysis. The same strategy was applied to search for articles on HCC recurrence with the key words “HBV” and “hepatocellular carcinoma recurrence”. The searches were limited to studies conducted in humans, and no language restrictions were imposed. Furthermore, the reference lists of retrieved articles were also reviewed to identify the additional studies. We conducted the meta-analysis and reported its results following the standard criteria [45].

The following criteria were applied into the literature selection for the dose-response meta-analysis on HBV DNA level and HCC risk: (1) the study had a case-control, nested case-control or cohort design; (2) the investigators assessed the relationship between multiple quantitative categories of HBV DNA level and risk of HCC; (3) the number of cases, the total subjects or person-years and corresponding odds radios (ORs), RRs or hazard ratios (HRs) together with 95% confidence interval (CI) for each category were provided. Studies on recurrence of HCC were included in another meta-analysis if they satisfied the following criteria: (1) the study had a cohort study design; (2) the study of interest was serum HBV load; (3) the overcome of interest was the incidence of HCC recurrence; (4) the RR estimates with 95% CIs were reported or available to be calculated.

### Data extraction

Data were extracted independently by two reviewers and cross-checked to resolve any discrepancies. The following information were extracted from each study: first author, publication year, study location, number of cases, sample size or person-years, categories of HBV DNA level, relevant RR and 95% CI, and covariates adjusted for in the multivariable analysis.

For each study, the median or mean HBV DNA level for every category was assigned to corresponding relative risk. When the median or mean value per category was not provided in the article, we assigned the midpoint of the upper and lower boundary in each category as the average level. If the upper or lower boundary was open-ended, we assumed that the boundary had the same amplitude as the adjacent category.

### Statistical analysis

All of HBV DNA levels were log_10_-transformed to normalize and unify the data and the quantitative unit was copies/ml. If the reported unit was IU/ml, we assumed that one IU was approximately equivalent to five copies according to the current World Health Organization HBV standard and consensus [46]. RR and the corresponding 95% CI were calculated to estimate the effect of HBV DNA level on HCC. Since the morbidity of HCC is relatively low among general population, the OR extracted from case-control studies is pretty close to RR. Thus, we reported all results as RR for briefness.

We conducted a dose-response meta-analysis of the association between HBV DNA level and HCC risk, using the method for trend estimation proposed by Greenland and Longnecker [47]. To examine the potential nonlinear relationship between them, we created restricted cubic splines with three knots at percentiles 10%, 50% and 90% of the distribution. A probability value for nonlinearity was calculated by testing the null hypothesis that the coefficient of the second spline was equal to zero. Between-study heterogeneity was evaluated by the *I*^2^ statistic (*I^2^* < 30%, no between-study heterogeneity or marginal heterogeneity; *I^2^* = 30%–70%, mild heterogeneity; *I^2^* > 70%, notable heterogeneity) [48]. A fixed effect model (Mantel-Haenszel method) was applied if heterogeneity was negligible, and random effect model (DerSimonian and Laird method) if heterogeneity was significant [49]. We conducted the overall meta-analysis and stratified analysis according to relevant covariates. In order to assess the influence of single data set on the whole estimate, we performed sensitivity analysis by omitting each study at a time. In addition, the publication bias was evaluated by Egger's test. All statistical analyses were conducted by Stata version11 and a two-tailed *P* < 0.05 was considered to be statistically significant.
